# Supplementary material for: Soliton confinement in a quantum circuit
Source: Nat Commun. 2023 Nov 16;14:7433. doi: 10.1038/s41467-023-43107-3 (PMC10654717; doi:10.1038/s41467-023-43107-3)
Supplement: Supplementary file 1 — Supplementary Information [file 41467_2023_43107_MOESM1_ESM.pdf]

Supplementary Information

Ananda Roy[1,*] and Sergei L. Lukyanov[1,†]

[1] *Department of Physics and Astronomy, Rutgers University, Piscataway, NJ 08854-8019 USA*

The supplementary material is organized as follows. In Sec. I, the scaling limit of the quantum electronic circuit array is described, starting with the free-boson model, followed by the sine-Gordon (sG) and the perturbed sG (psG) models. The computation of the two-point correlation functions for the sG model is given in Sec. I B. In Sec. II, results for the ground state energy and the mass of the lightest particle in the psG model are presented. In Sec. III, some details of the density matrix renormalization group simulations, together with more numerical results obtained for the lattice models, are given. Finally, in Sec. IV, generalization of this approach is described which leads to psG models where the parent sG model has n-fold degenerate minima with $n > 2$.

I. SCALING LIMIT OF THE QEC LATTICE HAMILTONIAN

A. Free Boson Model

We start with a variant of the Bose-Hubbard model [1, 2] governed by the lattice Hamiltonian

$$H_0 = E_c \sum_{k=1}^{L} n_k^2 + \epsilon E_c \sum_{k=1}^{L} n_k n_{k+1} - E_J \sum_{k=1}^{L} \cos(\phi_k - \phi_{k+1}) - E_g \sum_{k=1}^{L} n_k \tag{I.1}$$

with[1]

$$\big[n_j, \mathrm{e}^{\pm \mathrm{i}\phi_k}\big] = \pm\delta_{jk}\ \mathrm{e}^{\pm\mathrm{i}\phi_k}\ .$$

The system has been analyzed analytically in Ref. [3] (see also Ref. [4] for the $\epsilon = 0$ case) and numerically using the DMRG technique in Ref. [5]. For $E_J \ll E_c$, it can be either in a Mott-insulating phase with integer filling or in a charge-density-wave phase with half-integer filling (see Appendix B of Ref. [5] for the phase-diagram). Here, we focus on the case when the insulating phase has a density of Cooper-pairs is 1/2. For $\epsilon = 0.2$, this is achieved by tuning the gate voltage $E_g/E_c = 1.2$. In this case, as E_J/E_c increases from zero, for $E_J/E_c > 0.09$, the system becomes critical and its low-energy properties can be explored within conformal field theory (see Ref. [5] for details).

In the case of a critical lattice system subject to (quasi) periodic boundary conditions, conformal invariance predicts a so-called tower structure for the low-energy spectrum [6]. Each conformal tower is labeled by a pair of conformal dimensions $(\Delta, \bar\Delta)$ while excitations inside the tower are characterized by a non-negative integers $(N, \bar N)$. Denoting the excitation energy over the ground state by ΔE and the corresponding momenta by P, the energy-momentum spectrum of states from the conformal tower is described as

$$\Delta E \ = \ \frac{2\pi E_c}{L}\ u\ \big(\Delta + \bar\Delta + N + \bar N\big) + o(1/L) \tag{I.2}$$

$$P \ = \ \frac{2\pi E_c}{L}\,(\Delta - \bar\Delta + N - \bar N). \tag{I.3}$$

Assuming that ϵ and E_g/E_c are fixed as above, the "Fermi velocity" u is a certain function of dimensionless ratio E_J/E_c [see Supplementary Figure 3(a)].

The field theory underlining the critical behavior of the lattice system [Eq. (I.1)] is the Gaussian model defined by the Euclidean action

$$\mathcal{A}_0 = \frac{1}{2\pi K}\int d^2x\ \big[\,(\partial_\tau\phi)^2 + (\partial_x\phi)^2\,\big]\ . \tag{I.4}$$

In the condensed matter terminology, K is called the Luttinger parameter, which is a function of E_J/E_c [see Supplementary Figure 4(b)]. Since the length scale $1/E_c$ can be naturally identified with the lattice spacing, the integer k labeling the lattice sites in Eq. (I.1) and "physical" time t are related to the Euclidean coordinates (x,τ) as

$$x = k/E_c\ , \qquad \tau = -\mathrm{i}\,ut/E_c\ . \tag{I.5}$$

---

* ananda.roy@physics.rutgers.edu

† sergeil@physics.rutgers.edu

[1] In what follows we use a system of units with $\hbar = c = 1$, so that all energy scales have dimension $[\text{length}]^{-1}$.

B. The sine-Gordon model

Consider a perturbation of the Hamiltonian in Eq. (I.1):

$$H_1 = H_0 - E_{J_2} \sum_{k=1}^{L} \cos(2\phi_k). \tag{I.6}$$

The corresponding (renormalized) Euclidean action takes the form

$$\mathcal{A}_{\rm sG} = \int d^2x \left[\frac{1}{16\pi}\, \partial_\nu \varphi \partial^\nu \varphi - 2\mu\, \cos(\beta\varphi) \right]. \tag{I.7}$$

Here, instead of the Luttinger parameter K, we use the dimensionless coupling constant

$$\beta = \sqrt{K/2},\ \ 0 < \beta < 1 \tag{I.8}$$

and

$$\varphi = \phi\ \sqrt{8/K}\ . \tag{I.9}$$

The lattice energy scale E_c plays the role of the cut-off energy, while E_{J_2} in Eq. (I.6) is a bare coupling constant. In the scaling limit, $E_c \to \infty$ while the combination $E_{J_2} E_c^{1-2\beta^2}$ is kept fixed. To assign a precise meaning to the renormalized coupling μ, one should imposed a normalization condition on the renormalized field $\cos(\beta\varphi)$. The later has the scale dimension $d = 2\beta^2$ and is normalized by the condition

$$\lim_{r\to 0} r^{2d} \big\langle \cos(\beta\varphi)(x_1) \cos(\beta\varphi)(x_2) \big\rangle = \frac{1}{2} \qquad \big(r \equiv |x_1 - x_2|\big). \tag{I.10}$$

Then,

$$\mu = C\ E_{J_2} E_c^{1-2\beta^2}\ , \tag{I.11}$$

where the constant C depends on the dimensionless coupling β (see also Supplementary Figure 4). A remarkable feature of the model of Eq. (I.6) is that the scaling behavior occurs already for $E_{J_2}/E_c \lesssim 1$ for all $\beta^2 \in (0,1)$. To illustrate this, we consider two-point correlation functions of exponential fields.

Short distance expansion

Among local fields of the sine-Gordon model, a special role belongs to the exponential operators $\mathrm{e}^{\mathrm{i}\alpha\varphi}$. The short distance expansion of the two-point correlator of such fields was discussed in Ref. [7]. However, results of this work for the correlation function

$$\mathcal{G}_{\alpha_1,\alpha_2}(r) = \langle\, \mathrm{e}^{\mathrm{i}\alpha_1\varphi}(x_1)\ \mathrm{e}^{\mathrm{i}\alpha_2\varphi}(x_2)\,\rangle \tag{I.12}$$

can be applied literally for $\alpha_1 + \alpha_2 \neq 0$ only. Here, we are focusing on the case when $\alpha_2 = -\alpha_1 = \alpha$. The required small-$r$ expansion can be obtained from Eq. (2.23) from Ref. [7] through a certain limiting procedure. Below, we describe few leading terms of the short distance expansion of

$$\mathcal{G}_{-\alpha,\alpha}(r) = \langle\, \mathrm{e}^{-\mathrm{i}\alpha\varphi}(x_1)\ \mathrm{e}^{\mathrm{i}\alpha\varphi}(x_2)\,\rangle\ , \tag{I.13}$$

which is applicable to the case

$$|\alpha| \le \beta$$

for generic values of $\beta \in (0,1)$. Similar to Eq. (I.10), we fix the normalization of the exponential fields by the condition

$$\lim_{r\to 0} r^{4\alpha^2} \mathcal{G}_{-\alpha,\alpha}(r) = 1.$$

Then it is possible to show that

$$r^{4\alpha^2}\mathcal{G}_{-\alpha,\alpha}(r) = 1 + J(2\alpha\beta, -2\beta^2)\ \mu^2 r^{4(1-\beta^2)} - \tfrac{1}{4}\,\alpha^4\,\mathcal{H}(0)\ r^4 +\ \mu r^2\ \mathcal{G}_\beta \times \bigg[2S(2\alpha\beta) - (4\alpha\beta)^2\pi\ \log(r) - (2\alpha\beta)^2\pi\,\frac{\partial}{\partial\beta^2}\log\big(\mathcal{G}_\beta\big)\Big|_\mu\bigg] + O\Big(r^6, r^{8(1-\beta^2)}, r^{4(1+\beta^2)}\Big)\bigg] \ . \tag{I.14}$$

The formula calls for some remarks. Firstly, the symbol for the remaining term stands for

$$O(r^a, r^b, r^c) \equiv O(r^d) \quad \text{where} \quad d = \min(a,b,c) - \epsilon \quad \forall\epsilon > 0\ .$$

For explanation of the terms in Eq. (I.14), we refer the reader to Refs. [7, 8]. The expansion coefficients read explicitly:

(i)

$$J(a,c) = A(a,c) + 2B(a,c) + C(a,c) \tag{I.15}$$

with

$$A(a,c) \;=\; \big(1-\cos(\pi c)\cos(\pi(2a+c)\big) \times \Big[a\Gamma(1-a)\Gamma(1+a+c)\Gamma(-1-c)\,{}_3F_2^2(-c,-1-c,1-a;-a-c,2;1)\Big]^2\ ,$$

$$B(a,c) \;=\; \frac{a^2\,\pi^2\,\Gamma(1+c)\,\Gamma(-1-a-c)}{(1+a+c)(2+a+c)\Gamma(-a)}\ {}_3F_2\big(-c,-1-c,1-a;-a-c,2;1\big) \times\ {}_3F_2(a,1+a,2+c;2+a+c,3+a+c;1)$$

and

$$C(a,c) \;=\; -\frac{\Gamma(-1-a-c)\Gamma(-2-a-c)}{\Gamma(2+a+c)\Gamma(3+a+c)} \times \left[\frac{\Gamma(1+a)}{\Gamma(-a)}\ \frac{\pi\Gamma(2+c)}{\Gamma(-c)}\ {}_3F_2(a,1+a,2+c;2+a+c,3+c+a;1)\right]^2 .$$

Here ${}_3F_2(a_1,a_2,a_3;c_1,c_2;z)$ is the conventional generalized hypergeometric function at $z=1$. It should be pointed out that the definition (I.15) is literally applicable for $|\alpha| < \beta$. The individual terms in its r.h.s. diverges as $|\alpha| \to \beta$. Nevertheless the sum remains finite at $|\alpha| = \beta$.

(ii)

$$S(a) = -a^2\pi\,\big[\psi(1+a) + \psi(1-a) + 2\gamma_{\rm E} - 2\big]\ ,$$

where $\psi(z) = \partial_z \log\Gamma(z)$ and $\gamma_{\rm E}$ stands for the Euler constant.

(iii) Functions

$$\mathcal{G}_\beta = \frac{1}{2}\ (1+\xi)\ \frac{\Gamma(\frac{\xi}{2})}{\Gamma(1-\frac{\xi}{2})}\ \frac{\Gamma(\frac{1}{2}-\frac{\xi}{2})}{\Gamma(\frac{1}{2}+\frac{\xi}{2})}\ \left(\frac{\Gamma(1-\beta^2)}{\Gamma(\beta^2)}\right)^{1+\xi}\ (\pi\mu)^\xi$$

and

$$\mathcal{H}(0) = -\frac{16}{\pi^{\frac{3}{2}}\,\Gamma^3(\frac{3}{2}+\frac{\xi}{2})}\left[\frac{\Gamma(\frac{\xi}{2})}{\Gamma(1-\frac{\xi}{2})}\ \frac{\Gamma(\frac{1}{2}-\frac{\xi}{2})}{\Gamma(\frac{1}{2}+\frac{\xi}{2})}\right]^2\left(\frac{\Gamma(1-\beta^2)}{\Gamma(\beta^2)}\right)^{2(1+\xi)}\ (\pi\mu)^{2+2\xi}\ .$$

In the last formulae we use the so-called renormalized coupling constant which is related to β as

$$\xi = \frac{\beta^2}{1-\beta^2}\ . \tag{I.16}$$

Long distance expansion

The correlation function in Eq. (I.13) admits the large distance expansion in terms of the exact form-factors [9, 10]. The leading term corresponds to the zero-particle contribution, i.e., a vacuum expectation value of the exponential fields:

$$\lim_{r\to+\infty}\mathcal{G}_{-\alpha,\alpha}(r)=\mathcal{G}_\alpha^2\ ,\qquad \mathcal{G}_\alpha=\langle \mathrm{e}^{\pm\mathrm{i}\alpha\phi}\rangle\ .$$

The latter was found in Ref. [11],

$$\mathcal{G}_\alpha=\left[\pi\mu\ \frac{\Gamma(1-\beta^2)}{\Gamma(\beta^2)}\right]^{\frac{\alpha^2}{1-\beta^2}}\exp\left(\int_0^\infty\frac{\mathrm{d}t}{t}\ \left(\frac{\sinh^2(2\alpha\beta t)}{2\sinh(\beta^2t)\sinh(t)\cosh\big((1-\beta^2)t\big)}-2\alpha^2\,\mathrm{e}^{-2t}\right)\right)\ . \tag{I.17}$$

The particle spectrum of the sG model contains a soliton-antisoliton doublet with the same mass M. A relation between M and the dimensionfull coupling μ from the renormalized action Eq. (I.7) is known after the work [12],

$$\mu=\frac{\Gamma(\beta^2)}{\pi\Gamma(1-\beta^2)}\ \left[\frac{\sqrt{\pi}\Gamma(\frac{1}{2}+\frac{\xi}{2})}{2\Gamma(\frac{\xi}{2})}\ M\right]^{2-2\beta^2}\ . \tag{I.18}$$

Here, together with β^2, we use again the renormalized coupling ξ of Eq. (I.16).

If the dimensionless coupling β is restricted to the domain

$$\tfrac{1}{2}\le\beta^2<1\ ,$$

all the particles repell each other and no bound states are formed. In this case, the two leading terms corresponding to zero- and two- particle contributions to the correlator in Eq. (I.13) can be written in the form

$$\mathcal{G}_{-\alpha,\alpha}(r)=\mathcal{G}_\alpha^2\left(1+\frac{1}{2\pi^2}\ \int_{-\infty}^{+\infty}\mathrm{d}\theta\ |f_\alpha(\theta+\mathrm{i}\pi)|^2\ \cosh\left(\tfrac{\theta}{\xi}\right)\ K_0\Big(2Mr\cosh(\tfrac{\theta}{2})\Big)+\ldots\right)\ ,$$

where ovals stand for the four- and higher-particle contributions. The analytical form of $f_\alpha(\theta)$, which is a meromorphic function of the rapidity variable θ, was presented (in slightly different notations) in the work [13]. It is considerably simpler for $\alpha=\beta$ and $\alpha=\beta/2$ than for generic values of α [10, 13]:

$$f_\beta(\theta)=\tfrac{2}{\xi}\ \cot\left(\tfrac{\pi\xi}{2}\right)\ \frac{\theta\cosh(\frac{\theta}{2})}{\sinh(\frac{\theta}{\xi})}\ \exp\left[-\int_0^\infty\mathrm{d}t\ \frac{\sin^2\left(\frac{t\theta}{\pi}\right)}{t\sinh(2t)}\ \left(\frac{\sinh\big(t(\xi-1)\big)}{\cosh(t)\sinh(t\xi)}-2\ \mathrm{e}^{-2t}\right)\right]\ ,$$

while

$$f_{\beta/2}(\theta)=\frac{\theta\coth(\frac{\theta}{2})}{\xi\sinh(\frac{\theta}{\xi})}\ \ \exp\left[-\int_0^\infty\mathrm{d}t\ \frac{\sin^2\left(\frac{t\theta}{\pi}\right)}{t\sinh(2t)}\ \left(\frac{\sinh\big(t(\xi-1)\big)}{\cosh(t)\sinh(t\xi)}-2\ \mathrm{e}^{-2t}\right)\right]\ .$$

Here $K_\nu(x)$ is the conventional modified Bessel function of the second kind.

In the parameter domain

$$0<\beta^2<\tfrac{1}{2},$$

the soliton-antisoliton pair form bounded states – the breathers. The mass of the lightest breather, m_b, is simply related to the soliton mass:

$$m_b=2M\ \sin\left(\tfrac{\pi\xi}{2}\right)\ .$$

In the case

$$\tfrac{1}{3}<\beta^2<\tfrac{1}{2}\ ,$$

this is the only bound state and the one-particle contribution to the correlation function $\mathcal{G}_{-\alpha,\alpha}(r)$ is given by

$$\mathcal{G}^{(1-\text{part})}_{-\alpha,\alpha}(r) = \left[\frac{2\sin(\pi\alpha\sqrt{\xi(1+\xi)})}{\pi\sqrt{\xi(1+\xi)}}\right]^2 Z\ K_0(m_b r)\ .$$

Here Z is the so-called wave-function renormalization constant [14]

$$Z = \frac{\pi\xi(1+\xi)}{2\sin(\frac{\pi\xi}{2})}\ \exp\left(-\int_0^{\pi\xi}\frac{\mathrm{d}t}{\pi}\ \frac{t}{\sin(t)}\right)\ .$$

A comparison of the analytical predictions with the DMRG results is presented in Figure 2 of the main text.

C. The perturbed sine-Gordon model

Now, consider the Hamiltonian

$$H_2 = H_1 - E_{J_1}\sum_{k=1}^{L}\cos(\phi_k)\ = H_0 - E_{J_2}\sum_{k=1}^{L}\cos(2\phi_k) - E_{J_1}\sum_{k=1}^{L}\cos(\phi_k). \tag{I.19}$$

The corresponding action takes the form

$$\mathcal{A}_{\text{psG}} = \int d^2x\ \left[\frac{1}{16\pi}\ \partial_\nu\varphi\partial^\nu\varphi - 2\mu\ \cos(\beta\varphi) - 2\lambda\cos(\beta\varphi/2)\right], \tag{I.20}$$

where the field $\cos(\beta\varphi/2)$ with scale-dimension $\beta^2/2$ is normalized similarly as in Eq. (I.10). In terms of the bare couplings of the Hamiltonian in Eq. (I.19),

$$\lambda = C'\ E_{J_1}E_c^{1-\beta^2/2}, \tag{I.21}$$

where C' is a function of E_{J_1}/E_{J_2} and the Luttinger parameter. We use the dimensionless parameter

$$\eta = \frac{E_{J_1}/E_c}{(E_{J_2}/E_c)^\nu}, \qquad \nu = \frac{1-\beta^2/4}{1-\beta^2}. \tag{I.22}$$

This is expressed in terms of the renormalized couplings as:

$$\eta = \frac{C'}{C^\nu}\frac{\lambda}{\mu^\nu}, \tag{I.23}$$

where C and C' are β-dependent constants [see Eqs. (I.11, I.21)].

II. GROUND STATE ENERGY AND MASS OF THE LIGHTEST PARTICLE IN THE PERTURBED SINE-GORDON MODEL

In this section, we present the results for the ground state energy and the mass of the lightest particle in the psG model and compare with analytical predictions.

A. Ground state energy

The change in ground state energy density $\mathcal{E}$ of the psG model with respect to the sG model, is given, in general, by

$$\mathcal{E}_{\text{psG}} - \mathcal{E}_{\text{sG}} = M^2 G(\eta), \tag{II.1}$$

where the function $G(\eta)$ admits an expansion in a Taylor series in $\eta \sim \lambda$. To leading order in the perturbation theory,

$$\mathcal{E}_{\text{psG}} - \mathcal{E}_{\text{sG}} = -2\lambda\mathcal{G}_{\beta/2} + O(\lambda^2), \tag{II.2}$$

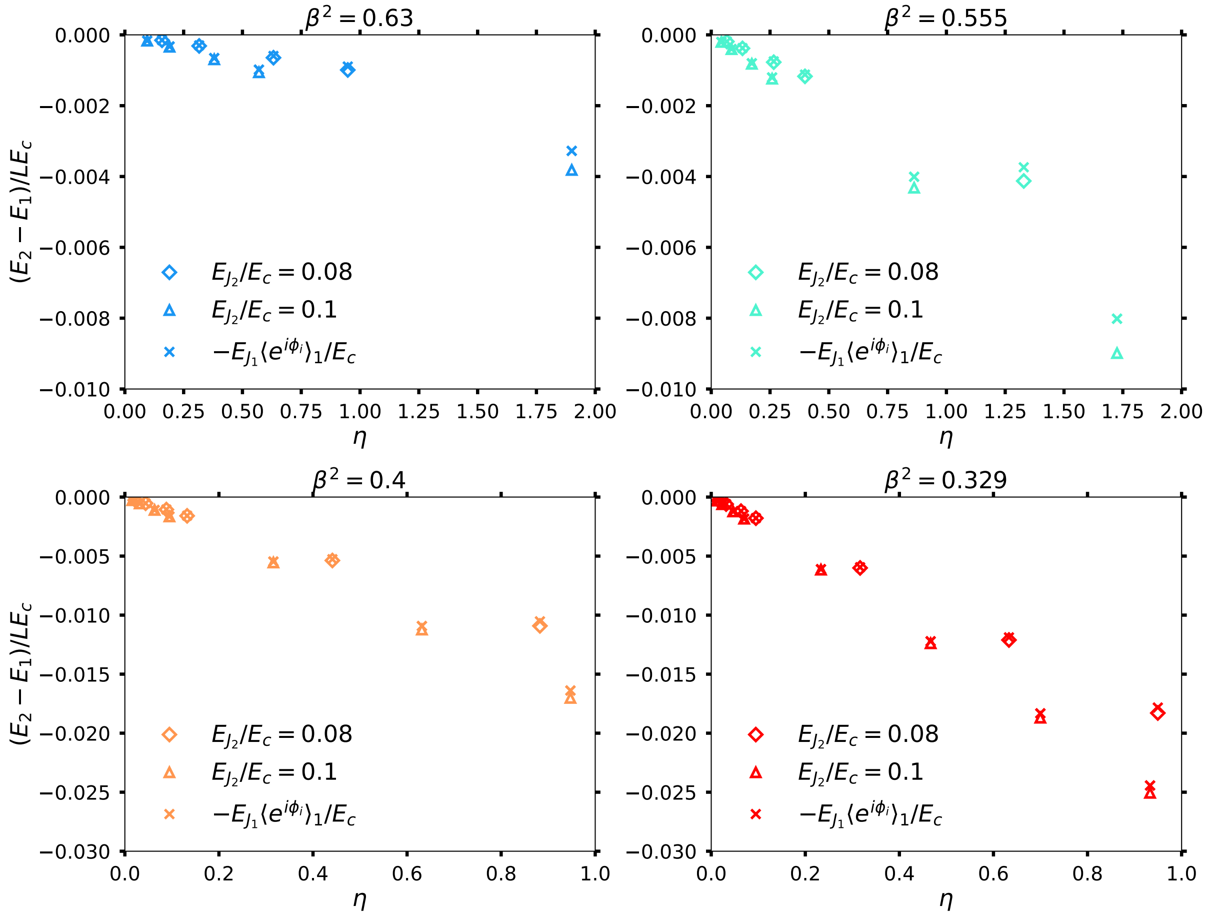

Supplementary Figure 1. Comparison of the change in the ground state energy density for the lattice model in Eq. (I.19) with respect to that in Eq. (I.6) for different choices of β^2 as a function of the dimensionless parameter η [see Eq. (I.22)]. The triangular markers correspond to different choices of lattice coupling E_{J_2}/E_c. The corresponding analytical predictions are shown as crosses.

where $\mathcal{G}_{\beta/2}$ is the expectation value of the operator $\mathrm{e}^{\mathrm{i}\beta\varphi/2}$, given by Eq. (I.17). For the lattice model, the corresponding first-order correction to energy, in units of E_c, is given by

$$\frac{E_2 - E_1}{LE_c} = -\frac{E_{J_1}}{E_c}\langle \mathrm{e}^{\mathrm{i}\phi_i}\rangle_1 + O(E_{J_1}^2/E_c^2), \tag{II.3}$$

where the expectation value is taken in the ground state of H_1. A comparison of this prediction with DMRG results for the ground state energy for the lattice model of Eq. (I.19) is shown in Supplementary Figure 1. Expectedly, the DMRG results are compatible with the perturbative prediction for smaller values of η.

B. Correction to the mass of the lightest sG breather

The $\cos(\beta\varphi/2)$ perturbation of the psG model induces corrections to the masses of the breathers that exist in the original sG model for $\beta^2 < 1/2$. To the first perturbative order, the difference between the breather mass $\tilde{m}_b$ in the psG model and that in the sG model is:

$$\frac{\tilde{m}_b - m_b}{M} = D\lambda + O(\lambda^2) \tag{II.4}$$

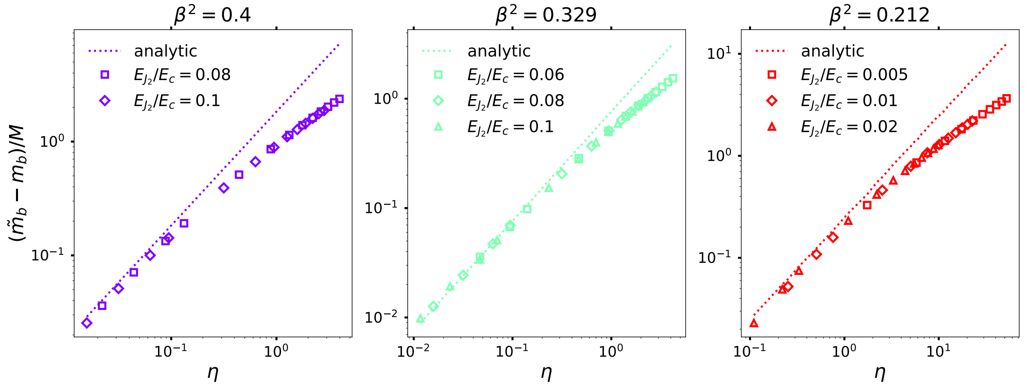

Supplementary Figure 2. Comparison of the analytical predictions computed within the one-particle approximation with the DMRG computation as a function of η [Eq. (I.22)] for three choices of β^2.

and the coefficient D was computed within "one-particle approximation" in Ref. [15]. The formula given in Eq. (5.13) of that paper can be simplified to

$$D \approx \frac{2\mathcal{G}_{\beta/2}}{M^2 \cos(\pi\xi/2)}, \tag{II.5}$$

where ξ, M are defined in Eq. (I.16) and Eq. (I.18) respectively. In terms of the lattice parameters, the leading-order correction to the breather mass can be rewritten as

$$\frac{\tilde{m}_b - m_b}{M} \approx \frac{1}{u^3 \cos(\pi\xi/2)} \left(\frac{E_c}{M}\right)^2 \frac{E_{J_1}}{E_c} \langle \mathrm{e}^{\mathrm{i}\phi_i} \rangle_1. \tag{II.6}$$

A comparison of this analytical result (dotted line) with our numerical data (squares, diamonds and triangles) is given in Supplementary Figure 2. The lowest-order predictions work well for $\beta^2 \ll 1/2$ even for $\eta \sim 1$. As β^2 approaches 1/2, it is expected that the corrections from the higher particle contributions become important. Nevertheless, the agreement between the DMRG and analytical results is still reasonable.

C. Scaling of the mesons masses in the perturbed sine-Gordon model

For $\beta^2 = 1/2$, the psG model is equivalent to the non-interacting, massive Dirac fermion theory, perturbed by the $\cos(\beta\varphi/2)$ potential. For this case, following Ref. [16] (see also Refs. [17, 18]), the masses of the mesons can be approximately computed using the quantum mechanical Hamiltonian for the soliton and antisoliton pair:

$$H_{\mathrm{NI-2p}} = \frac{p_1^2}{2M} + \frac{p_2^2}{2M} + \sigma|x_1 - x_2|. \tag{II.7}$$

Here, M is soliton mass and $\sigma = 2\lambda\langle e^{i\beta\varphi/2}\rangle$ is the string tension within this approximation [see Figure 3 of main text for numerical results for σ]. The masses of the mesons, to leading order, are determined by the zeros of the Airy function z_n, $n = 1, 2, \ldots$:

$$m_n = 2M + \left(\frac{\sigma^2}{M}\right)^{1/3} z_n. \tag{II.8}$$

In terms of the dimensionless parameter η [see Eq. (I.22)], for small η, one has

$$\frac{m_n - 2M}{M} \propto \eta^{2/3} \qquad (\eta \ll 1). \tag{II.9}$$

Strictly-speaking, this formula holds true only for $\beta^2 = 1/2$. In general, the small-η behavior is described by an exponent α that depends on β (see Figure 4 of the main text). The computation of this exponent is an interesting open problem which would require inclusion of the soliton-antisoliton interaction.

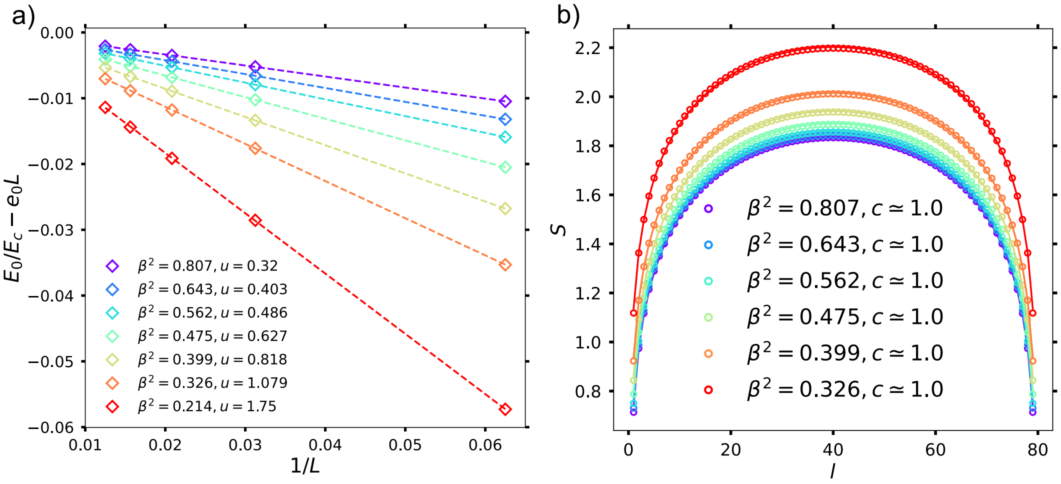

Supplementary Figure 3. DMRG results for the Fermi velocity (u, see panel a) and the central charge (c, see panel b) for the free boson model.

III. NOTES ON DMRG SIMULATION

Next, we provide details of the numerical simulations performed using DMRG, building on the setup developed in Refs. [5, 19].

At each lattice site, we define a quantum rotor in the occupation basis: $\{|n_k\rangle\}$, where n_k-s range from -8 to $+8$ in steps of 1. This leads to a local Hilbert space with dimension 17. This truncation was sufficient to get the accurate and stable data for the half-filling case analyzed in this work. Note that the Hilbert space is larger than that of the earlier works. This is because of the existence of the $\cos(2\phi_k)$ term in Eq. (I.1), which necessitates a doubling of the local Hilbert space dimension. The lattice vertex operators can be straightforwardly defined based on their action on the basis states [19]. The simulations were done using the single-site DMRG routine of the TeNPy package [20].

The free boson model

To characterize the free-boson phase, we set $E_{J_1} = E_{J_2} = 0$ and $E_g/E_c = 1.2$ [see discussion below Eq. (I.1)]. The Luttinger parameter K is computed by calculating the two-point correlation function of the $\mathrm{e}^{i\phi_k}$ operator in the ground-state obtained using infinite DMRG technique [see Figure 2(a) of the main text]. The velocity of light, u, is obtained by computing the scaling of the ground-state energy with system size using the conformal field theory prediction for periodic boundary conditions [6]:

$$\frac{E_0}{E_c} = e_0 L - \frac{\pi c u}{6L} + o(1/L), \tag{III.1}$$

where the two terms on the r.h.s respectively correspond to the extensive and Casimir contributions to the energy. The central charge c is independently verified to be 1 by using the scaling of the entanglement entropy with the subsystem size [21, 22]:

$$S(l) = \frac{c}{3} \ln\left[\frac{L}{\pi} \sin\left(\frac{\pi l}{L}\right)\right] + S_0, \tag{III.2}$$

where l is the subsystem size, S_0 is a non-universal contribution to the entanglement entropy. We compute the Fermi velocity by varying the system size as $L = 16p$, $p = 1, 2, \ldots, 5$ [see Supplementary Figure 3(a)]. The corresponding results for the entanglement entropy are shown in Supplementary Figure 3(b).

The sine-Gordon model

The ground state of the Hamiltonian H_1, [Eq. (I.6)], was computed by conserving the $\mathbb{Z}_2$ charge associated with the parity of the Cooper-pair number: $P = \prod_{j=1}^{L} \mathrm{e}^{\mathrm{i}\pi n_j}$. Let the two eigenstates with parity eigenvalues ± 1 be denoted

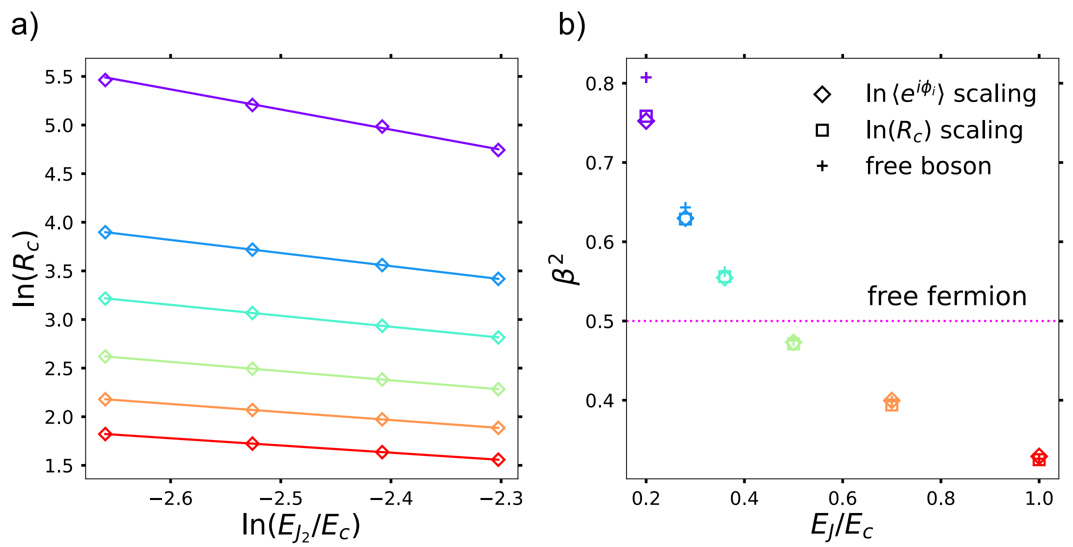

Supplementary Figure 4. DMRG results for the variation of the correlation length R_c with the coupling E_{J_2}/E_c (panel a) and the comparison with the values of $\beta^2 = K/2$ obtained form the Luttinger parameter K of the free boson theory and the scaling of the vertex operator $\mathrm{e}^{\mathrm{i}\phi_k}$ (panel b, see also Figure 2 of the main text). Note that the results for the correlation length, together with Eqs. (I.18, III.3), provide a numerical estimate of the non-universal constant C of Eq. (I.11).

by $|\psi_\pm\rangle$. For each of these states, $\langle\psi_\pm|\mathrm{e}^{\mathrm{i}\phi_j}|\psi_\pm\rangle = 0$ since the operator $\mathrm{e}^{\mathrm{i}\phi_j}$ change the parity sector. Diagonalizing $P_{ab} = \langle\psi_a|P|\psi_b\rangle$, where $a, b = \pm$, gives the eigenstates of $\mathrm{e}^{\mathrm{i}\phi_j}$, which become the symmetry-broken ground states of the sG model in the scaling limit.

To minimize finite size effects in the computation of the correlation functions, the infinite DMRG technique was used. To obtain the soliton mass, the correlation length R_c of the matrix product state was computed. The numerical results for R_c were converged to the three significant figures. In the parameter domain $1/4 < \beta^2 < 1$, the soliton mass is expressed in terms of the correlation length as

$$M = \frac{E_c}{uR_c}. \tag{III.3}$$

For $\beta^2 < 1/4$, in the above formula, M should be replaced by the mass of the lightest bound state. In the scaling limit,

$$R_c \propto \left(\frac{E_{J_2}}{E_c}\right)^{-1/(2-2\beta^2)}. \tag{III.4}$$

The dependence of R_c with the ratio E_{J_2}/E_c is depicted in Supplementary Figure 4. From this, one can extract the value of the dimensionless coupling β^2. A comparison of the values of β^2 so-obtained with those found using the other methods is shown in Supplementary Figure 4(b) (see also Figure 2 of the main text).

The perturbed sine-Gordon model

To obtain the results for the psG model, the Hamiltonian in Eq. (I.19) with $E_{J_1}, E_{J_2} \neq 0$ was analyzed. For the computation of the string tension, a soliton-antisoliton pair is created one lattice site apart by applying the soliton creating operators, described in the main text. The expectation value of the lattice Hamiltonian in the state with the soliton-antisoliton pair one lattice site apart was taken as a reference energy.

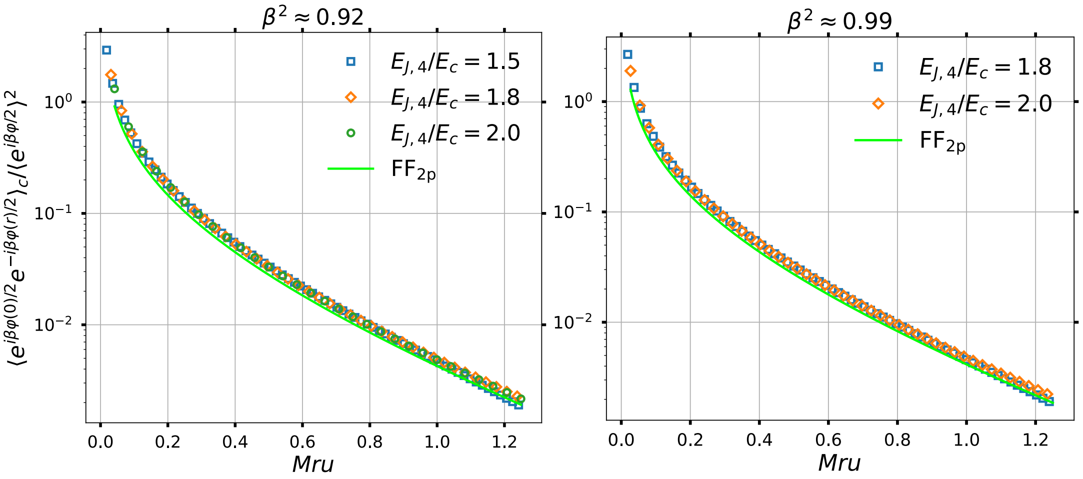

Supplementary Figure 5. Results for the correlation functions for the sG model with four-fold degenerate minima for $\beta^2 \approx 0.92$ and $\beta^2 \approx 0.99$. The numerical results are shown for the ground state of the Hamiltonian in Eq. (IV.1) with $n = 4$. The markers are DMRG data obtained for the corresponding values of the lattice couplings, while the solid line is the form-factor computation including the two-particle contributions. The near-perfect scaling collapse for $1 - \beta^2 \ll 1$ is a consequence of the low corrections to scaling for the QEC model.

IV. GENERALIZATIONS TO SINE-GORDON MODELS WITH $n > 2$-FOLD DEGENERATE MINIMA AND THEIR PERTURBATIONS

The QEC proposed in this work can be straightforwardly generalized to give rise to psG models with n-fold degenerate minima, where $n > 2$. To that end, consider the Hamiltonian:

$$H_1^n = H_0 - E_{J_n} \sum_{k=1}^{L} \cos(n\phi_k), \tag{IV.1}$$

where H_0 is defined in Eq. (I.1). This Hamiltonian, in the scaling limit, gives rise to an sG model with n-fold degenerate minima, the latter being at $2p\pi/n$, $p = 0, 1, \dots, n-1$. The corresponding Euclidean action is given by Eq. (I.7), but now the value of sG coupling β^2 is related to the Luttinger parameter K as

$$\beta^2 = \frac{n^2 K}{8}. \tag{IV.2}$$

In this case, the $\cos\beta\varphi$ potential is relevant for $0 \le K \le 8/n^2$. The corresponding $\cos n\phi$ Josephson junction can be realized by starting with $\cos\phi$ and $\cos 2\phi$ Josephson junction and recursively applying the scheme of Ref. [23]. The corresponding psG models can be realized by considering perturbations of the lattice Hamiltonian of Eq. (IV.1) given by:

$$H_2^{n,m} = H_1^n - E_{J_m} \sum_{k=1}^{L} \cos(m\phi_k), \tag{IV.3}$$

where we choose $m < n$ for definiteness. The case analyzed in the main text corresponds to the case $n = 2, m = 1$. Note that such n-fold degenerate sG models could be constructed starting with the XXZ spin chain and introducing interactions that involve nonlocal interactions involving spins that are next-to-nearest neighbors or are further apart. However, these spin chains suffer from larger corrections to scaling than the QEC lattice model (see discussion in Ref. [24]).

Next, we present DMRG results for certain properties of the model of Eq. (IV.1) for $n = 4$. Since $\beta^2 = 2K$ in this case, it suffices to consider the case when $K \in [0, 1/2]$ for the free theory (when $E_{J_4} = 0$). The corresponding phase occurs in the Bose-Hubbard model of Eq. (I.1) for both integer and half-integer filling. In order to consider the simplest lattice model, we consider the case of zero filling with $\epsilon = 0$. This is obtained by choosing $E_g/E_c = 0$ (see Appendix B of Ref. [5]).

Supplementary Figure 5 compares the two-point correlation functions of the vertex operator $e^{i\beta\varphi/2}$ obtained using DMRG and form-factor (Sec. I B) computations including two-particle contributions. The results are shown for $\beta^2 \approx$

0.92 and $\beta^2 \approx 0.99$. The same for $\beta^2 < 0.8$ are similar to what is shown in Figure 2 of the main text and are not shown for brevity. The near-perfect scaling collapse for $1-\beta^2 \ll 1$ indicates the low-corrections to scaling for the QEC model, making the latter a particularly suitable candidate for the analysis of the sG model near the Kosterlitz-Thouless point. The psG model describing the scaling limit of Eq. (IV.3) with $n=4, m=2$ was analyzed using DMRG. Results obtained are similar to the $n=2, m=1$ case and are not shown for brevity.

Before concluding, we remark that perturbations of the sG model with n-fold degenerate minima can be used to systematically give rise to multicritical Ising models [25]. The main idea behind the QEC realization of multicritical Ising models is based on the well-known notion that the diagonal, unitary minimal models of conformal field theories arise as multicritical points of an effective Ginzburg-Landau action [26]. To arrive at this effective action, we consider perturbations of the euclidean quantum sG action with p-fold degenerate minima describing the scalar field φ:

$$\mathcal{A} = \int d^2x \left[\frac{1}{16\pi}(\partial_\nu\varphi)^2 - 2\mu\cos(\beta\varphi)\right] - \sum_{n=1}^{p-1} 2\lambda_n \int d^2x \cos\left(\frac{n\beta\varphi}{p} + \delta_n\right), \tag{IV.4}$$

where μ, λ_n-s are coupling constants and δ_n-s are suitably chosen phases. The case $\lambda_n = 0\ \forall\ n$ corresponds to the ordinary sG model with p-fold degenerate minima with coupling constant β, where we consider the case $0 \le \beta^2 \le 1$. Appropriate choices of $\{\lambda_n, \delta_n\}$ induces a flow to the quantum critical points of multicritical Ising universality class. The latter are characterized by the central charges

$$c_p = 1 - \frac{6}{(p+1)(p+2)},\ p = 2, 3, \ldots. \tag{IV.5}$$

The corresponding QEC Hamiltonians are given by [25]

$$H = E_c \sum_{j=1}^{L} n_j^2 + \epsilon E_c \sum_{j=1}^{L} n_j n_{j+1} - E_g \sum_{j=1}^{L} n_j - E_J \sum_{j=1}^{L} \cos(\phi_j - \phi_{j+1}) - \sum_{n=1}^{p}\sum_{j=1}^{L} E_{J_n}\cos(n\phi_j + \delta_n), \tag{IV.6}$$

where δ_p is set to 0. These Hamiltonians have been numerically analyzed in Ref. [25] to capture signatures of Ising and tricritical Ising models in QEC lattices.

[1] M. P. A. Fisher, P. B. Weichman, G. Grinstein, and D. S. Fisher, Boson localization and the superfluid-insulator transition, Phys. Rev. B **40**, 546 (1989).
[2] S. Sachdev, *Quantum Phase Transitions* (Cambridge University Press, 2011).
[3] L. I. Glazman and A. I. Larkin, New quantum phase in a one-dimensional josephson array, Phys. Rev. Lett. **79**, 3736 (1997).
[4] R. M. Bradley and S. Doniach, Quantum fluctuations in chains of josephson junctions, Phys. Rev. B **30**, 1138 (1984).
[5] A. Roy, F. Pollmann, and H. Saleur, Entanglement Hamiltonian of the 1+1-dimensional free, compactified boson conformal field theory, J. Stat. Mech. **2008**, 083104 (2020), arXiv:cond-mat/2004.14370.
[6] P. Francesco, P. Di Francesco, P. Mathieu, D. Sénéchal, and D. Senechal, *Conformal Field Theory*, Graduate Texts in Contemporary Physics (Springer, 1997).
[7] V. Fateev, D. Fradkin, S. Lukyanov, A. Zamolodchikov, and A. Zamolodchikov, Expectation values of descendent fields in the sine-gordon model, Nucl. Phys. B **540**, 587 (1999).
[8] B. Doyon and S. Lukyanov, Fermion schwinger's function for the SU(2)-thirring model, Nucl. Phys. B **644**, 451 (2002).
[9] B. Berg, M. Karowski, and P. Weisz, Construction of green's functions from an exact s-matrix, Phys. Rev. D **19**, 2477 (1979).
[10] F. Smirnov, *Form Factors in Completely Integrable Models of Quantum Field Theory*, Advanced series in mathematical physics (World Scientific, 1992).
[11] S. L. Lukyanov and A. B. Zamolodchikov, Exact expectation values of local fields in quantum sine-Gordon model, Nucl. Phys. B **493**, 571 (1997), arXiv:hep-th/9611238.
[12] A. B. Zamolodchikov, Mass scale in the sine-gordon model and its reductions, Int. J. Mod. Phys. **10**, 1125 (1995).
[13] S. L. Lukyanov, Form-factors of exponential fields in the sine-Gordon model, Mod. Phys. Lett. A **12**, 2543 (1997), arXiv:hep-th/9703190.
[14] M. Karowski and P. Weisz, Exact form-factors in (1+1)-dimensional field theoretic models with soliton behavior, Nucl. Phys. B **139**, 455 (1978).
[15] Z. Bajnok, L. Palla, G. Takacs, and F. Wagner, Nonperturbative study of the two frequency sine-Gordon model, Nucl. Phys. B **601**, 503 (2001), arXiv:hep-th/0008066.
[16] B. M. McCoy and T. T. Wu, Two-dimensional ising field theory in a magnetic field: Breakup of the cut in the two-point function, Phys. Rev. D **18**, 1259 (1978).

[17] P. Fonseca and A. Zamolodchikov, Ising field theory in a magnetic field: Analytic properties of the free energy, J. Stat. Phys. **110**, 527 (2003).
[18] P. Fonseca and A. Zamolodchikov, Ising spectroscopy. I. Mesons at $T < T_c$, (2006), arXiv:hep-th/0612304.
[19] A. Roy, J. Hauschild, and F. Pollmann, Quantum phases of a one-dimensional majorana-bose-hubbard model, Phys. Rev. B **101**, 075419 (2020).
[20] J. Hauschild and F. Pollmann, Efficient numerical simulations with Tensor Networks: Tensor Network Python (TeNPy), SciPost Phys. Lect. Notes , 5 (2018).
[21] C. Holzhey, F. Larsen, and F. Wilczek, Geometric and renormalized entropy in conformal field theory, Nucl. Phys. B **424**, 443 (1994).
[22] P. Calabrese and J. Cardy, Entanglement entropy and quantum field theory, J. Stat. Mech: Theory and Experiment **2004**, P06002 (2004).
[23] P. Brooks, A. Kitaev, and J. Preskill, Protected gates for superconducting qubits, Phys. Rev. A **87**, 052306 (2013).
[24] A. Roy, D. Schuricht, J. Hauschild, F. Pollmann, and H. Saleur, The quantum sine-Gordon model with quantum circuits, Nucl. Phys. B **968**, 115445 (2021), arXiv:quant-ph/2007.06874.
[25] A. Roy, Quantum Electronic Circuits for Multicritical Ising Models, (2023), arXiv:2306.04346 [quant-ph].
[26] A. B. Zamolodchikov, Conformal Symmetry and Multicritical Points in Two-Dimensional Quantum Field Theory. (In Russian), Sov. J. Nucl. Phys. **44**, 529 (1986).
